# COVID-19 mRNA Vaccination, ABO Blood Type and the Severity of Self-Reported Reactogenicity in a Large Healthcare System: A Brief Report of a Cross-Sectional Study

**DOI:** 10.7759/cureus.20810

**Published:** 2021-12-29

**Authors:** Jennifer D Allan, Daniel McMillan, Marc L Levi

**Affiliations:** 1 Anesthesiology, University of North Carolina at Chapel Hill, Chapel Hill, USA

**Keywords:** vaccination, reactogenicity, large healthcare system, covid-19, abo blood type

## Abstract

Introduction

It has been anecdotally observed that ABO blood type may have an impact on the severity of the side-effects experienced by those receiving mRNA vaccination for COVID-19.

Methods

As part of a larger study, a retrospective cross-sectional survey was made available to approximately 33,000 front-line healthcare workers, students and volunteers who were offered voluntary vaccination in a state-wide healthcare system during phase one of the state’s vaccine roll-out. A secondary endpoint of the survey was to determine if there was any relationship between vaccination reactogenicity and ABO blood type.

Results

4009 responses were received - a 12.15% response rate. 3700 respondents answered the blood type question, and of those, 2878 knew their blood type. By Kruskal-Wallis test, there was no statistically significant association between any blood type and any side effect for either of the COVID-19 mRNA vaccines.

Conclusions

COVID-19 mRNA vaccination may cause significant reactogenicity. However, ABO blood type does not appear to be a predictor of vaccine reactogenicity.

## Introduction

On December 31, 2019, the World Health Organization (WHO) was first informed of a series of cases of pneumonia of unknown etiology in the city of Wuhan, China [1,2]. On February 11, 2020, the WHO officially announced that the causative agent for this novel illness was a coronavirus. The disease vector, formally named SARS-CoV-2 and the disease it causes, COVID-19, has become responsible for over 4 million deaths worldwide at the time of this writing [3], rapidly becoming the worst public health crisis since the influenza pandemic of 1918 [4].

Messenger RNA (mRNA) vaccines for COVID-19 were introduced rapidly due in large part to existing research by National Institutes of Health staff at the Vaccine Research Center and the preparation of “prototype coronavirus vaccines” to a generic coronavirus [5]. While the development of the technology and knowhow to manufacture mRNA vaccines has been quietly progressing for many years, the public’s reception of the Pfizer and Moderna vaccines for COVID-19 has been mixed due to their unfamiliarity with the technology, as well as a lack of awareness regarding both the research pathways and the side-effects of the vaccines. While significant vaccine reactogenicity was seen in preclinical trials, there was much speculation on what would account for the variation in reactogenicity seen between individuals. Shortly after mRNA COVID-19 vaccines by Pfizer [6] and Moderna [7] became available in December 2020, it was anecdotally observed that individuals of different ABO blood types appeared to experience different degrees of reactogenicity to vaccination. The frequency and severity of the reactogenicity seemed to correlate with their blood type, whereby individuals with blood type A had more severe side effects than those with blood group O.

Associations between ABO blood groups and the risk of SARS-CoV-2 infection have been documented in the literature [8,9]. The literature is very limited, however, on any relationship between vaccine reactogenicity itself and the ABO blood type. Check et al published the results of a small observational study of 91 individuals where they showed a statistically significant self-reported incidence of worse vaccine reactogenicity in individuals with type A blood as compared to those with type O [10]. As part of a larger project evaluating COVID-19 vaccination and work-related absences, this project aimed to further elucidate this relationship beyond the initial anecdotal observations in a population of front-line healthcare workers employed by or working at a large, multi-site, state-wide, tertiary medical system who were offered the mRNA vaccination as part of the phase one vaccine roll-out.

## Materials and methods

After Institutional Review Board review and waiver, a survey was made available to employees, healthcare providers, and volunteers of a state-wide tertiary care system through a link published in staff and medical provider electronic newsletters. Vaccination for the target population began December 14, 2020, and continued through March 2021. The retrospective data collection began on March 16, 2021, and continued through May 4, 2021. The target population was comprised of approximately 33,000 individuals with patient-facing responsibilities that made them eligible for voluntary COVID-19 vaccination with the aforementioned mRNA vaccines during the early vaccination period. 

Applying a cross-sectional study design, a self-reporting questionnaire (Appendices) was used to collect anonymous information. As pertaining to this secondary study objective, the questions asked included blood type, the type of vaccination received, the reactogenicity experienced, and the intensity and duration of symptoms for each injection of the two-shot mRNA vaccine. The symptoms queried were based on a modified version of the list published by the FDA on the websites describing mRNA vaccines [11,12]. Patients reported severity on a modified Likert scale where zero indicated they did not experience the symptom and ten was considered incapacitating.

## Results

**Figure 1:**
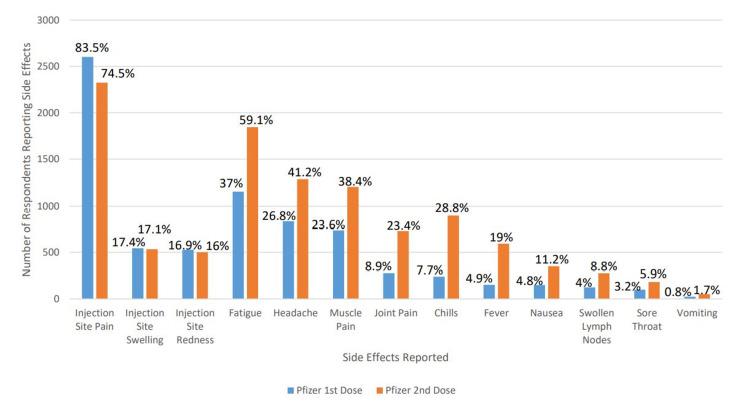
Side Effects Seen With Pfizer Vaccination

**Figure 2:**
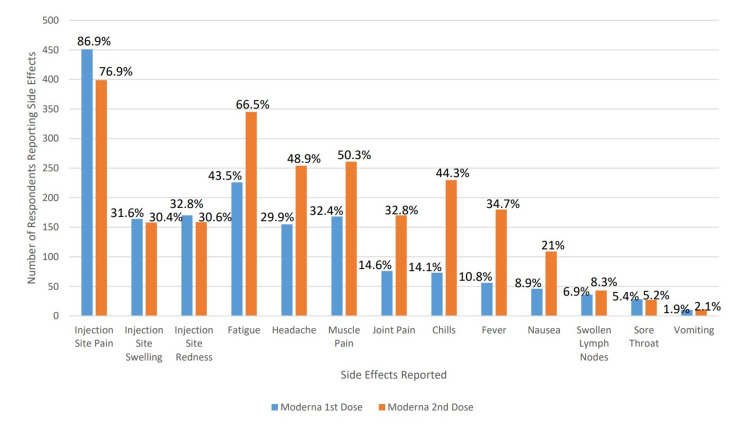
Side Effects Seen With Moderna Vaccination

A total of 4,009 responses were recorded out of approximately 33,000 eligible individuals, a 12.15% response rate. Not all respondents answered all questions. The basic demographic information of the respondents is noted in Table 1. Of the 4,009 respondents, 3,638 reported they had received the vaccine. The side-effects reported by the vaccinated individuals, by vaccine type and scheduled vaccine are reported in Figures 1, 2. 3,700 participants answered the blood type question and 2,878 individuals knew their blood type and were vaccinated. By Kruskal-Wallis test, there was no statistically significant association between any reported blood type and any side effect for either dose of either vaccine product. The data regarding the reported side effects as stratified by blood type are reported in Tables 2, 3.

**Table 1:** Demographic Information ^1^Statistics presented: n (%)

Characteristic	N = 4,009^1^
Gender	
Female	3,418 (86%)
Male	534 (13%)
Other	5 (0.1%)
Unknown	52
Age	
18 - 24	156 (4.0%)
25 - 34	839 (21%)
35 - 44	920 (23%)
45 - 54	1,032 (26%)
55 - 64	828 (21%)
65 - 74	169 (4.3%)
75 - 84	2 (<0.1%)
Unknown	63
Education	
Associate degree (e.g. AA, AS)	663 (17%)
Bachelor's degree (e.g. BA, BS)	1,323 (34%)
Doctorate or professional degree (e.g. MD, DDS, PhD)	551 (14%)
High school degree or equivalent (e.g. GED)	142 (3.6%)
Less than a high school diploma	2 (<0.1%)
Master's degree (e.g. MA, MS, MEd)	765 (20%)
Some college, no degree	477 (12%)
Unknown	86
Job	
Advanced Practice Provider	201 (5.1%)
Attending Physician	197 (5.0%)
Nurse	1,137 (29%)
Other	1,147 (29%)
Resident or Fellow Physician	102 (2.6%)
Student	73 (1.9%)
Support Staff	718 (18%)
Technician	341 (8.7%)
Volunteer	4 (0.1%)
Unknown	89
American Indian or Alaska Native	67 (1.7%)
Asian	238 (5.9%)
Black or African American	434 (11%)
Hispanic or Latino	148 (3.7%)
Native Hawaiian or Pacific Islander	6 (0.1%)
White	3,113 (78%)
Other	55 (1.4%)

**Table 2:** The Impact of Blood Type on Reactogenicity With the First Dose of Vaccine ^1^Statistics presented: Mean (SD); ^2^Statistical tests performed: Kruskal-Wallis test

			Blood Type						
Side Effect	Overall, N = 3,700^1^	A, N = 995^1^		AB, N = 148^1^	B, N = 369^1^	O, N = 1,366^1^		Unknown, N = 822^1^	p-value^2^
Injection site pain	3.8 (2.83)	3.8 (2.75)		3.7 (2.79)	3.4 (2.81)	3.9 (2.85)		3.8 (2.91)	0.092
Injection site redness	0.7 (1.79)	0.7 (1.83)		0.6 (1.67)	0.6 (1.77)	0.7 (1.81)		0.7 (1.74)	>0.9
Injection site swelling	0.7 (1.85)	0.7 (1.81)		0.6 (1.78)	0.6 (1.65)	0.8 (1.92)		0.7 (1.86)	0.7
Fatigue	1.8 (2.87)	1.8 (2.80)		1.7 (2.78)	1.6 (2.85)	1.8 (2.85)		1.9 (3.00)	0.4
Headache	1.3 (2.53)	1.3 (2.49)		0.9 (2.06)	1.3 (2.74)	1.3 (2.54)		1.3 (2.55)	0.5
Muscle pain	1.2 (2.44)	1.2 (2.39)		0.9 (2.12)	1.2 (2.59)	1.1 (2.37)		1.3 (2.60)	0.2
Chills	0.5 (1.73)	0.5 (1.71)		0.4 (1.39)	0.6 (2.09)	0.4 (1.55)		0.5 (1.90)	0.2
Joint pain	0.5 (1.83)	0.5 (1.82)		0.3 (1.53)	0.6 (1.95)	0.5 (1.81)		0.5 (1.87)	0.6
Fever	0.3 (1.38)	0.3 (1.43)		0.2 (0.85)	0.4 (1.64)	0.3 (1.29)		0.3 (1.40)	0.5
Nausea	0.3 (1.25)	0.3 (1.21)		0.2 (1.13)	0.3 (1.45)	0.2 (1.25)		0.2 (1.21)	0.7
Vomiting	0.0 (0.60)	0.0 (0.51)		0.1 (0.84)	0.1 (0.89)	0.0 (0.56)		0.0 (0.55)	0.11
Swollen lymph nodes	0.2 (1.25)	0.2 (1.17)		0.3 (1.46)	0.2 (1.24)	0.2 (1.23)		0.3 (1.32)	>0.9
Sore throat	0.1 (0.91)	0.1 (0.97)		0.1 (0.63)	0.1 (0.93)	0.1 (0.81)		0.2 (1.00)	0.2

**Table 3:** The Impact of Blood Type on Reactogenicity With the Second Dose of Vaccine ^1^Statistics presented: Mean (SD); ^2^Statistical tests performed: Kruskal-Wallis test

			Blood Type						
Characteristic	Overall, N = 3,700^1^	A, N = 995^1^		AB, N = 148^1^	B, N = 369^1^	O, N = 1,366^1^		Unknown, N = 822^1^	p-value^2^
Injection site pain	3.1 (2.84)	3.1 (2.74)		2.9 (2.79)	2.8 (2.79)	3.2 (2.83)		3.1 (3.00)	0.048
Injection site redness	0.7 (1.93)	0.7 (1.88)		0.3 (0.96)	0.7 (1.97)	0.8 (1.99)		0.8 (2.01)	0.3
Injection site swelling	0.8 (2.01)	0.8 (1.99)		0.4 (1.17)	0.7 (2.03)	0.8 (2.05)		0.8 (2.06)	0.2
Fatigue	3.4 (3.49)	3.5 (3.45)		3.0 (3.30)	3.1 (3.46)	3.5 (3.53)		3.3 (3.51)	0.2
Headache	2.2 (3.12)	2.2 (3.05)		1.7 (2.71)	2.0 (3.05)	2.2 (3.20)		2.3 (3.14)	0.3
Muscle pain	2.3 (3.28)	2.3 (3.24)		1.7 (2.82)	2.1 (3.23)	2.3 (3.31)		2.2 (3.34)	0.2
Chills	1.9 (3.20)	1.9 (3.23)		1.2 (2.50)	1.8 (3.24)	1.9 (3.22)		1.9 (3.20)	0.3
Joint pain	1.5 (2.95)	1.6 (2.98)		1.2 (2.68)	1.3 (2.80)	1.5 (3.00)		1.5 (2.96)	0.6
Fever	1.2 (2.64)	1.2 (2.70)		0.6 (1.88)	1.0 (2.43)	1.2 (2.69)		1.2 (2.69)	0.059
Nausea	0.6 (1.93)	0.6 (1.98)		0.3 (1.39)	0.5 (1.63)	0.6 (1.93)		0.7 (2.06)	0.11
Vomiting	0.1 (0.87)	0.1 (0.94)		0.1 (0.52)	0.1 (0.88)	0.1 (0.83)		0.1 (0.92)	>0.9
Swollen lymph nodes	0.5 (1.77)	0.5 (1.85)		0.3 (1.44)	0.4 (1.69)	0.5 (1.85)		0.4 (1.60)	0.5
Sore throat	0.3 (1.27)	0.2 (1.19)		0.1 (0.66)	0.3 (1.52)	0.3 (1.21)		0.3 (1.42)	0.2

## Discussion

**Figure 3:**
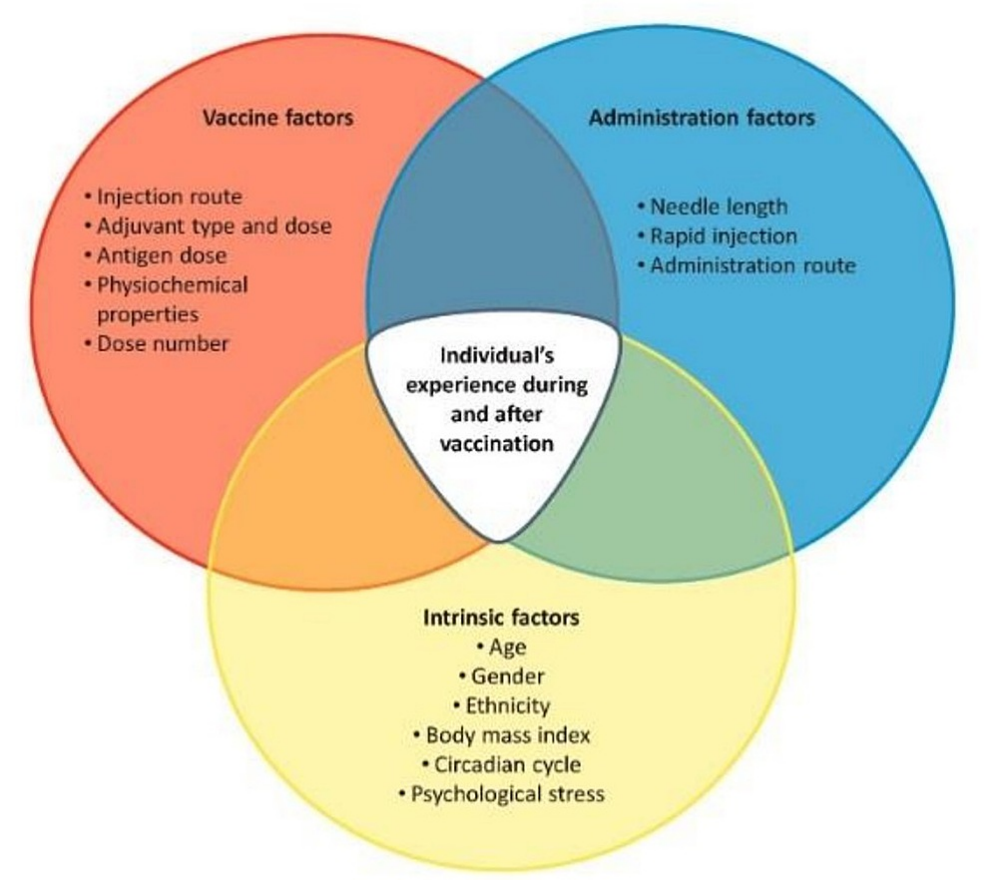
Summary of Vaccination and Host Factors That can Influence Reactogenicity With permission of the author [14].

In December 2020, the CDC Advisory Committee on Immunization Practices recognized that COVID-19 vaccination was expected to elicit local and systemic reactogenicity [13]. Patient-reported adverse effects can be impacted by many factors, including age, gender, general health, ethnicity, vaccine formulation, route of administration, injection technique and individual psychological stresses (Figure 3) [14]. As such, it is challenging to assess the direct impact of any individual demographic factor on the severity and duration of vaccine reactogenicity. 

ABO blood group antigens are expressed widely on cells throughout the body, and the importance of ABO membrane antigens extends beyond transfusion medicine [15]. The seroconversion to the live, attenuated influenza vaccine has been documented to be dependent on ABO blood group, whereby individuals of blood type A seroconverted after receiving a single dose, as opposed to two doses required for other ABO blood types [16]. It has also been reported that individuals with type A blood demonstrate an increased odds of COVID-19 infection while individuals with type O blood demonstrate a decreased odds of infection in [17,18]. It would therefore seem plausible for there to be an association between ABO blood grouping and vaccine reactogenicity. 

As previously cited, Check et al demonstrated a statistically significant association between ABO blood group and self-reported symptom severity whereby individuals with type A blood reported more severe reactogenicity than those with blood group O [10]. Although the methodology we employed for rating severity was different from that employed by Check et al, we were unable to support their findings. While ABO blood type may have an effect on the severity of COVID-19 infection and disease, we found no statistical correlation between ABO blood type and any reactogenicity symptoms with either vaccine.

This study has a number of weaknesses and limitations. The anecdotal observation that initially led to this study necessitated a retrospective data collection methodology whereby significant recall bias could be expected. Additionally, the study goals and methodology may have created a non-response/participation bias that limited the participation of individuals who did not experience reactogenicity.

Finally, the sampling method, which relied on emailed newsletters, biased the respondent sample to those who opened the email and read the newsletters. This sampling bias, as well as the preponderance of nurses responding, may account for the overwhelming number of female respondents and those who describe their job type as “other.”

## Conclusions

Local and systemic reactogenicity are known and expected occurrences after the Pfizer and Moderna mRNA COVID-19 vaccinations and are seen in a considerable percentage of those surveyed. Although ABO blood type appears to be associated with COVID-19 disease severity, we found no connection between ABO blood type and vaccine reactogenicity with mRNA vaccines. Due to the timing of this study, we were unable to ascertain if a relationship exists between blood type and reactogenicity for other types of vaccines, specifically the Janssen/Johnson & Johnson vaccine, which uses a viral vector. Further research into this area is warranted.

## Appendices

COVID-19 Side Effects and Work-Related Absences Survey Questions

What is your gender?

o Female

o Male

o Other

What is your age?

o 18 - 24

o 25 - 34

o 35 - 44

o 45 - 54

o 55 - 64

o 65 - 74

o 75 - 84

o 85 or older

How would you describe yourself? Please select all that apply.

o American Indian or Alaska Native

o Asian

o Black or African American

o Other

o Native Hawaiian or Pacific Islander

o White

o Other

What is the highest degree or level of school you have completed?

o Less than a high school diploma

o High school degree or equivalent (e.g. GED)

o Some college, no degree

o Associate degree (e.g. AA, AS)

o Bachelor's degree (e.g. BA, BS)

o Master's degree (e.g. MA, MS, MEd)

o Doctorate or professional degree (e.g. MD, DDS, PhD)

What is your job description?

o Advanced Practice Provider

o Attending Physician

o Nurse

o Resident or Fellow Physician

o Technician

o Student

o Support Staff

o Volunteer

o Other

What is your PRIMARY place of employment?

o Caldwell UNC Health Care

o Chatham Hospital

o Johnston Health

o Nash UNC Health Care

o Oslow Memorial Health

o Pardee UNC Health Care

o Shared Services

o UNC Health Southeastern

o UNC Hillsborough Hospital

o UNC Hospitals--Chapel Hill

o UNC Lenoir Health Care

o UNC Physicians Network

o UNC REX

o UNC Rockingham Health Care

o UNC School of Medicine

o Wayne UNC Health Care

o Other

What was your enthusiasm to vaccination PRIOR to being offered the vaccination? (Zero is totally unenthusiastic, 10 is absolutely thrilled)

0

1

2

3

4

5

6

7

8

9

10

Have you EVER had a positive diagnosis of COVID-19?

o YES

o No

Did you opt to receive the vaccination?

o Yes

o No

What is your blood type?

o A

o B

o O

o AB

o Unknown

What is your weight (pounds)?

0

50

100

150

200

250

300

350

400

450

500

What is your height (inches)?

0

10

20

30

40

50

60

70

80

90

100

Which COVID-19 Vaccination manufacture's product did you receive?
 

o Pfizer

o Moderna

Were you SCHEDULED to work the day after your FIRST vaccination?

o Yes

o No

Did you modify your work or vaccination schedule for the FIRST vaccination dose in ANTICIPATION of potential side effects from the vaccination?

o Yes

o No

What side effects did you experience after your FIRST vaccination dose? (zero=none, ten=severe)

0

1

2

3

4

5

6

7

8

9

10

Injection site pain

Injection site redness

Injection site swelling

Fatigue

Headache

Muscle pain

Chills

Joint pain

Fever

Nausea

Vomiting

Swollen lymph nodes

Sore throat

Approximately how many days did the side effects of vaccination last after your FIRST vaccination dose?

0

1

2

3

4

5

6

7

8

9

10

How many days off work did you take after your FIRST vaccination? (Due to vaccination side effects OR on the advice of the COVID vaccination hot-line)

0

1

2

3

4

5

6

7

8

9

10

Were you SCHEDULED to work the day after your SECOND vaccination?

o Yes

o No

Did you modify your work or vaccination schedule for the SECOND vaccination dose in ANTICIPATION of potential side effects from the vaccination?

o Yes

o No

What side effects did you experience after your SECOND vaccination dose? (zero=none, ten=severe)

0

1

2

3

4

5

6

7

8

9

10

Injection site pain

Injection site redness

Injection site swelling

Fatigue

Headache

Muscle pain

Chills

Joint pain

Fever

Nausea

Vomiting

Swollen lymph nodes

Sore throat

Approximately how many days did the side effects of vaccination last after your SECOND vaccination dose?

0

1

2

3

4

5

6

7

8

9

10

How many days off work did you take after your SECOND vaccination? (Due to vaccination side effects OR on the advice of the COVID vaccination hot-line)

0

1

2

3

4

5

6

7

8

9

10

If you didn’t opt to receive the vaccine, please tell us briefly why you have chosen not to be vaccinated:

________________________________________________________________
